# Fetal MRI study of brain differences in early-onset fetal growth restriction versus healthy controls at 30 weeks of gestation

**DOI:** 10.1016/j.eurox.2025.100417

**Published:** 2025-07-03

**Authors:** Lotte Meijerink, Inge van Ooijen, Fieke Terstappen, Thomas Alderliesten, Rutger A.J. Nievelstein, Femke Lammertink, Manon Benders, Mireille Bekker

**Affiliations:** a Department of Obstetrics, Division Woman & Baby, University Medical Center Utrecht, Utrecht, Utrecht University, the Netherlands; b Department of Neonatology, Division Woman & Baby, University Medical Center Utrecht, Utrecht, Utrecht University, the Netherlands; c Department of Pediatric Radiology & Nuclear Medicine, Division Imaging & Oncology, University Medical Center Utrecht, Utrecht, Utrecht University, the Netherlands

**Keywords:** Fetal growth restriction, Magnetic resonance imaging, Fetal brain

## Abstract

**Objective:**

To identify volumetric and diffusion-related brain differences expressed as apparent diffusion coefficient (ADC) values between early-onset brain-sparing fetal growth restriction (FGR) and healthy controls using magnetic resonance imaging (MRI) at 30 weeks of gestation.

**Method:**

This prospective, observational, monocenter cohort study included singleton pregnancies with early-onset brain-sparing FGR at the University Medical Center Utrecht. FGR fetuses were compared to healthy controls from the Utrecht YOUth Cohort. Fetal MRI of the brain was performed including T2-weighted and diffusion weighted imaging (DWI) sequences. We measured 2D biometrics, 3D volumetrics using BOUNTI, and ADC values in multiple brain and placental regions. Values were corrected to 30 weeks of gestation.

**Results:**

The study included 26 FGR fetuses at gestational age (GA) 26.3–32 weeks and 71 controls at GA 30.1–34 weeks. At 30 weeks, total brain volume (TBV) was significantly smaller in FGR (144.2 ± 11.5 vs 166.9 ± 17.5 milliliters, p < 0.001). After dividing all absolute volumes by TBV, only cerebellar volume remained significantly reduced (0.045 [0.00] vs 0.048 [0.01], p = 0.006). ADC values were lower in all brain regions except the cerebellum. Placental ADC values were also significantly lower in FGR.

**Conclusion:**

Altered brain development in brain-sparing FGR is already present at 30 weeks of gestation. Lower brain volumes and ADC values may reflect the effects of altered perfusion, chronic hypoxia and microstructural changes in the brains of FGR fetuses. Future studies linking these MRI findings to long-term neurodevelopmental outcomes will aid in more personalized prognoses and might also inform the timing of delivery, ultimately enhancing clinical decision-making.

## Highlights

• At 30 weeks of gestation, total brain volume is significantly smaller in early-onset brain-sparing FGR compared to healthy controls.
• When adjusting for total brain volume, only cerebellar volume remains reduced in FGR.
• ADC values in multiple brain regions and the central and peripheral placenta are significantly lower in FGR.

## Introduction

Fetal growth restriction (FGR) is a condition in which the fetus does not reach its optimum growth potential often leading to significant perinatal morbidity and mortality [1]. The main cause of FGR in developed countries is placental insufficiency [2]. To protect the brain from hypoxemia and undernutrition due to this placental insufficiency, the fetus undergoes hemodynamic adaption called brain-sparing. In brain-sparing, fetal cardiac output in redistributed towards the brain through vasodilation of the middle cerebral artery (MCA) [3]. Although the term implies a protective mechanism, this adaptation is a marker of fetal compromise, and it is known that early-onset FGR neonates with antenatal brain-sparing have adverse neurocognitive outcomes [4], [5], [6], [7].

In-utero development might provide a window of opportunity for treatment, but the identification of fetuses at risk for adverse neurodevelopmental outcomes remains a challenge [8]. If FGR-specific abnormalities in fetal brain development are identified and linked to long-term neurodevelopmental outcomes, interventions could be targeted to prevent altered brain development in FGR, or timing of delivery can be adjusted to prevent impaired brain development.

Magnetic Resonance Imaging (MRI) enables structural, volumetric, and functional assessment of the fetal brain, complementing the existing golden standard of ultrasound [9], [10]. T2-weighted imaging allows for volumetric analysis of the fetal brain [11]. Additionally, diffusion-weighted imaging (DWI) provides insight into microstructure and hypoxic-ischemic damage by measuring the diffusion of water, expressed in apparent diffusion coefficients (ADC) [12], [13], [14], [15].

Previous studies have used MRI to assess fetal brain development in FGR reporting on volumetrics as well as ADC values [16]. Yet, control groups often include participants undergoing fetal MRI for clinical reasons and not healthy volunteers. Additionally, results are heterogeneous due to various methodologies (e.g. inclusion criteria or manual segmentation with lack of motion correction), small samples, different or no definitions of brain-sparing, and MRI frequently performed at a later gestational age (GA).

This study aims to identify whether structural and diffusion-related differences in the brain already exist at 30 weeks of gestation using MRI by comparing fetuses with early-onset brain-sparing FGR and healthy controls.

## Methods

### Study design and setting

This prospective, observational, monocenter cohort study included singleton pregnancies with early-onset brain-sparing FGR at the University Medical Center Utrecht from the 1st of June 2022 until the 1st of June 2024. Approval from the local ethical committee was obtained (protocol number 22–552, approved on the 7th of April 2022). Eligible pregnant women were counseled for this study upon hospital admission. Both parents provided written informed consent for inclusion.

### Participants

Inclusion criteria were singleton pregnancies with early-onset FGR defined as an estimated fetal weight (EFW) and/or abdominal circumference (AC) below the 10th percentile using the Hadlock formula with a GA between 24 and 32 weeks. Brain-sparing was defined as an umbilical artery (UA) Doppler pulsatility index (PI) above the 95th percentile in combination with middle cerebral artery (MCA) PI below the 5th percentile or a cerebroplacental ratio (CPR) < 1 [17], [18], [19]. Exclusion criteria were confirmed maternal or fetal infection, fetal genetic or congenital abnormalities and contraindications for MRI.

The control participants were derived from the YOUth cohort, a longitudinal healthy cohort including nearly 4000 pregnancies with postnatal follow-up until adulthood to investigate brain development [20]. The data-request was approved, published and fetal MRI data from the YOUth Baby MRI study was shared for 71 participants with normal biometry and Dopplers around 30 weeks of gestation [21].

### MRI protocol

A 3.0 T MRI (Philips Intera, Philips Healthcare Ltd, Best, The Netherlands) was performed as soon as brain-sparing was diagnosed and within the week after administering corticosteroids. Pregnant women were scanned without sedation in left lateral tilt position using a transmit coil. The standardized protocol included T2-weighted sequences with a voxel size of 1.25 × 1.25 mm, a slice thickness of 2.5 mm with a negative slice gap of −1.25, an echo time (TE) of 180 ms, and a repetition time (TR) 55321 ms. DWI was performed in the transversal plane with a voxel size of 2.25 × 2.25 mm, recon voxel size: 1.09 × 1.09, a slice thickness of 4 mm and no slice gap, a TE of 78 ms and TR of 2797 ms and included the b-values of 0 and 800. The total scan duration was 30 min.

### MRI analyses

All MRI scans were also checked for structural abnormalities by an experienced pediatric radiologist (RJN) to diminish interobserver variability. Fetal brain biometry was measured using the fetal dimensions tool by Kyriakopoulou and percentiles were used for analyses since these are already corrected for GA [22]. Fetal motion was corrected retrospectively using slice-to-volume registration (SVR) generating a 3D T2-weighted SVR image of the fetal brain. Thereafter, the BOUNTI method was applied to segment 19 brain regions [11]. The fetal brain segmentation quality was then assessed using ITK-SNAP [23]. We included the following volumes for our analyses: [1] cortical and white matter volumes (sum of left and right hemispheres), [2] cerebellar volume (sum of left cerebellar hemisphere, right cerebellar hemisphere and vermis), [3] deep gray matter volume (sum of left and right lentiform nucleus and thalamic volumes), and [4] total brain volume (TBV: sum cortex, white matter, brainstem, cerebellum and deep gray matter)

### Processing of ADC maps

The quality of the ADC maps of the fetal brain was ranked one to five by L.M. where one is considered poor quality and five optimal quality, from three onwards was considered diagnostic [24]. Participants were excluded if the MRI had a quality below two. The placement of the regions of interests (ROI) in the frontal white matter (FWM), occipital white matter (OWM), centrum semi ovale (CSO), thalami, cerebellar hemispheres (CBH), and pons was performed twice by the same researcher (LM) according to the methodology described by Arthurs et al. [12]. For the FWM, OWM, CSO and thalami the ROI surface was 30–60mm^2^ and for the CBH and pons this was 5–15mm^2^. The ROI was as large as possible, with the standard deviation of the pixels within ten percent otherwise the ROI was decreased in size manually. If this standard deviation was larger than ten percent at the smallest ROI, the measurement was excluded for analysis. The mean ADC value in the left and right hemisphere was used for analysis [12]. Intra-observer reliability analysis was conducted and the effect of the timing of corticosteroid administration was evaluated. Blinding to the clinical condition was not performed. However, to minimize potential bias, the standardized methodology mentioned above was applied for FGR and controls 4 weeks apart and in separate files.

If the largest surface area of the placenta was included in the DWI sequence, ADC values in the central and peripheral placenta were measured as described by Moradi et al. with two ROIs near the cord insertion and 2 ROIs more than two centimeter away from the placental edge [25]. The average of these two sites were analyzed separately due to potential differences in perfusion [25], [26].

### Statistical analysis

Data was analyzed using IBM SPSS Statistics Version 27.0 (IBM Corp., Armonk, NY). All data was tested for normality using the Shapiro-Wilk test [27]. The baseline characteristics were reported as the mean ± standard deviation (SD) for parametric continuous data and tested with an independent student’s T-test to compare fetuses with FGR to healthy fetuses. Non-parametric data was tested with a Mann-Whitney *U* test and reported as median with the interquartile range. Categorical dichotomous information was presented as number and percentage of the group and comparison for this between the two groups was analyzed by a Fisher’s exact test. Correlations were tested using Pearson correlation or a Spearman’s rank test depending on normality of the variable. Linear regression models were conducted to test the association between ADC-values in the fetal brain and the timing of corticosteroid administration in the FGR group. A two-sided *p*-value under 0.05 was considered statistically significant.

The difference in GA was corrected to 30 weeks for the volumetric segmentations and the ADC values since these measurements are gestational age dependent [28], [29]. Correction was done by using linear regression models for the FGR cohort and controls separately, acknowledging that brain growth may differ between these populations [30].Although fetal brain growth is nonlinear across gestation, between 24 and 34 weeks the growth curve appears to follow a linear trajectory [29]. A post hoc power analysis was conducted to contextualize observed findings by calculating Hedges g’ value for parametric variables and Cliff’s delta for nonparametric variables[31], [32], [33].

## Results

### Descriptive data

**Fig. 1:**
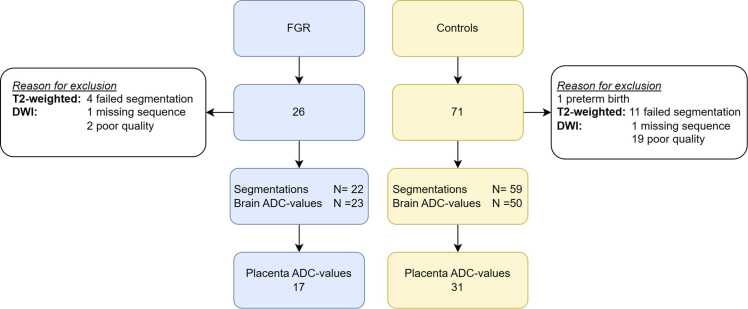
Flow Chart Showing The Selection of Participants.

In total 26 participants were included in the FGR cohort and 71 for the controls (Fig. 1**)**. The median time between CCS administration and MRI was 6 days (IQR 8). The mean quality for the diffusion scans was 3.1 ± 0.8 for FGR and 3.01 ± 0.6 for controls. The maternal ages were similar for FGR and the healthy controls (32.3 ± 4.8 vs 32.9 ± 3.2 years). A larger percentage of women smoked in the FGR group (11.5 % vs 1.4 %). In the FGR group 57.7 % were primiparous and 46.5 % in the control group. Of the fetuses 34.6 % were female in the FGR group compared to 57.7 % in the healthy control group (Table 1). Biometric and Doppler data from the FGR cohort can be found in Table S1.

**Table 1:** Baseline Characteristics of Study Participants: Brain Volumes, Diffusion-Weighted Imaging, and Total Cohort.

	Brain volumes			Diffusion-Weighted Imaging			Total		
	FGR (N = 22)	Control (N = 59)	p-value	FGR (N = 23)	Control (N = 50)	P-value	FGR (N = 26)	Control (N = 71)	p-value
Maternal age (years)	32.3 ± 4.5	32.8 ± 3.3	0.57	32.3 ± 5.1	33.0 ± 3.5	0.52	32.3 ± 4.8	32.9 ± 3.2	0.54
Maternal BMI (kg/m^2^)	26.1 ± 6.1	24.4 ± 5.1	0.21	25.4 ± 5.9	23.7 ± 5.1	0.23	25.9 ± 5.7	24.2 ± 4.9	0.16
Primiparity (%)	11 (50.0 %)	19 (40.4 %)	0.46	13 (56.5 %)	16 (40.0 %)	0.21	15 (57.7 %)	33 (46.5 %)	**0.02**
Caucasian (%)	12 (54.5 %)	58 (98.3 %)	**< 0.001**	14 (60.9 %)	49 (98 %)	**< 0.001**	15 (57.7 %)	71 (100 %)	**< 0.001**
Gender, female (%)	7 (31.8 %)	34 (57.6 %)	**0.04**	7 (30.4 %)	30 (60.0 %)	**0.02**	9 (34.6 %)	41 (57.7 %)	**0.04**
Maternal smoking (%)	3 (13.6 %)	1 (1.7 %)	**0.03**	3 (13.0 %)	1 (2.0 %)	0.05	3 (11.5 %)	1 (1.4 %)	**0.03**
GA at MRI (weeks)	29.9 ± 1.8	32.0 ± 1.3	**< 0.001**	29.5 ± 1.8	32.0 ± 1.2	**< 0.001**	29.7 ± 1.8	31.9 ± 1.2	**< 0.001**

BMI: Body mass index, kg: kilograms; DWI: Diffusion-weighted imaging; FGR: fetal growth restriction, GA: gestational age, MRI: magnetic resonance imaging.

### MRI Biometrics & Volumetrics

All 2D percentiles of the fetal brain biometry measurements on MRI were significantly smaller in the FGR group (Table 2). The mean TBV for FGR at 30 weeks of gestation was significantly lower compared to the control group, 144.2 ± 11.5 vs 166.9 ± 17.51 (p < 0.001). All regional brain volumes were significantly smaller for FGR (Table 3). There were no differences between the left and right hemispheres (Table S2). When correcting for TBV, only the cerebellar volumes remained significantly smaller for FGR (Table 3).

**Table 2:** Percentiles of Fetal Brain Biometry between FGR and control using MRI.

	FGR (N = 25)	Control (N = 41)	p-value
Head circumference	3.8 (15.9)	93.0 (43.0)	< 0.001
Transverse cerebellar diameter	6.3 (9.8)	37.5 (46.5)	< 0.001
Brain biparietal diameter	10.3 (28.4)	64.9 (47.6)	< 0.001
Brain fronto-occipital length	5.5 (20.9)	56.7 (58.6)	< 0.001
Skull biparietal diameter	3.7 (17.1)	93.1 (23.0)	< 0.001
Skull occipitofrontal diameter	8.5 (69.7)	87.1 (58.2)	< 0.001
Extra-cerebral CSF	13.3 (16.5)	95.0 (23.1)	< 0.001
Vermis height	3.5 (15.4)	39.8 (62.7)	< 0.001
Vermis Width	29.8 (76.4)	93.9 (37.8)	< 0.001
Atrial diameter	5.4 (40.1)	53.5 (58.7)	< 0.001

CSF: cerebrospinal fluid, FGR: fetal growth restriction

**Table 3:** Differences in absolute regional brain volumes (ml) and proportionally to the total brain volume (TBV)between early-onset brain-sparing FGR and controls at 30 weeks of gestation.

**Variable**	**FGR (N = 22)**	**Control (N = 59)**	**p-value**^**3**^
Cortex	38.96 ± 3.19	45.02 ± 5.49	< 0.001
White Matter	88.84 (8.49)	99.46 (17.52)	< 0.001
Brainstem	3.50 ± 0.27	3.95 ± 0.34	< 0.001
Cerebellum	6.49 ± 0.72	7.97 ± 0.98	< 0.001
Deep Gray Matter	7.65 ± 0.76	8.66 ± 0.82	< 0.001
**Total Brain Volume (TBV)**	**144.24 ± 11.54**	**166.87 ± 17.51**	**< 0.001**
Cortex / TBV	0.267 (0.02)	0.269 (0.02)	0.742
White Matter / TBV	0.613 (0.02)	0.608 (0.02)	0.378
Brainstem / TBV	0.024(0.00)	0.024 (0.00)	0.384
Cerebellum / TBV	0.045 (0.00)	0.048 (0.01)	**0.006**
Deep Gray Matter / TBV	0.053 (0.00)	0.052 (0.00)	0.161

FGR: fetal growth restriction, TBV: Total brain volume

### ADC values

The ADC values in the FWM, OWM, CSO, thalami and pons were significantly lower in the FGR group compared to healthy controls with the largest difference between the OWM and the thalami (Table 4). The intra-observer reliability scored excellent: 0.890, 0.902, 0.915, 0.959, 0.924, and 0.870 for the FWM, OWM, CSO, Thalami, CBH and pons, respectively. Timing of corticosteroids in FGR was not significantly associated with the GA-corrected ADC values of the fetal brain. Moreover, including corticosteroid timing alongside GA was also not associated with the uncorrected ADC values in the FWM, OWM, CSO, Thalami, CBH and pons. The placenta ADC values were significantly lower for FGR compared to healthy controls.

**Table 4:** ADC values of the fetal brain and placenta at 30 weeks of gestation.

			FGR (N = 23)	Control (N = 50)	p-value
Fetal brain		FWM	1.72 ± 0.16 (n = 22)	1.85 ± 0.15	0.002
		OWM	1.73 ± 0.11 (n = 21)	1.89 ± 0.15	< 0.001
		CSO	1.78 ± 0.15 (n = 22)	1.88 ± 0.15	0.010
		Thalami	1.20 (0.17) (n = 21)	1.34 (0.10)	< 0.001
		CBH	1.45 (0.23) (n = 22)	1.43 (0.16)	0.73
		Pons	1.12 (0.14)	1.26 (0.21)	0.001
Placenta		Central	1.15 (0.69)	1.79 (0.40)	< 0.001
		Peripheral	0.91 (0.81)	1.91 (0.49)	< 0.001

ADC: apparent diffusion coefficient in e-3mm^2/s; CBH: cerebellar hemispheres; CSO: centrum semiovale; FWM: frontal white matter; OWM: occipital white matter. Data is missing if region of interest was not representative, which was the case if the relative standard deviation of the pixels was > 10 % of the mean ADC value.

### Post Hoc Power Analysis

All absolute volumes demonstrated large effect sizes, indicating substantial differences between the groups given the sample sizes. In contrast, the ratios exhibited negligible differences with very small effect sizes except for the cerebellum/TBV, which show a modest effect. Regarding the ADC values, the OWM, thalami and placental regions showed strong effects (Table S3).

## Discussion

In this study we found significantly reduced total brain volumes and lower ADC values in the fetal brain for brain-sparing FGR corrected to 30 weeks of gestation using MRI. The observed reductions in brain volumes and ADC values may reflect the effects of altered cerebral hemodynamics and chronic hypoxia in FGR fetuses. All absolute regional brain volumes were significantly smaller for FGR fetuses. The ADC values for all brain regions were lower in early-onset FGR except for the cerebellum.

Despite using different methods often lacking 3-dimensional reconstruction, the reported reduced TBV for FGR is in line with previous studies [16]. The reduction in TBV might be attributed to chronic hypoxia leading to neuronal degeneration and hypomyelination [34], [35], [36], [37], [38], [39], [40], [41], [42]. The clinical relevance of smaller brain volumes and adverse neurocognitive outcomes has been established in preterm infants at term equivalent age, childhood, and adolescence [43]. Andescavage et al. illustrated in 28 infants how fetal brain volumes in FGR were positively associated with and self-regulation, movement quality, attention, and less lethargy and non-optimal reflexes [44].

After correcting all regional brain volumes for TBV, only cerebellar volume was reduced in brain-sparing FGR - a notable finding as the cerebellum is commonly used as a reliable estimator for the determination of GA late in pregnancy even in FGR [45], [46], [47], [48]. The altered fetal hemodynamics might negatively impact cerebellar volumes since FGR with Doppler abnormalities causes significantly smaller cerebellar volumes than without Doppler abnormalities [49]. Literature is conflicting on the cerebellar volume in FGR warranting further research to confirm our finding in other studies [16], [50].

The reduced ADC values in the brain and placenta are reported by previous studies at various GAs [16]. ADC values are a measure of water diffusion and can be reduced by microstructural changes or due to hypoxia and/or ischemia [12], [13], [14], [15]. We speculate that the included FGR-affected fetuses with brain-sparing are unable to fully compensate leading to chronic hypoxic-ischemic and microstructural changes in the developing fetal brain. Comparable to our results, Arthurs et al. showed lower ADC values in the FWM, CSO, thalami, and pons and no difference in cerebellum at 30 weeks GA [51]. Autopsies in their study revealed chronic hypoxia-related injuries and white matter cytotoxic edema as a response to acute ischemia reducing the diffusion of water and thus reducing ADC values [12]. The absence of a significant difference in ADC-values for the cerebellum, is in line with literature and could be potentially attributed to an underestimation of the actual cerebellar injury by ADC values. A postmortem study of infants with hypoxic ischemic encephalopathy (HIE) illustrated that cerebellar ADC values do not show the anticipated reductions that would fit the actual histopathological injury [52]. The clinical relevance of these reduced ADC values was demonstrated by one multicenter study in 56 small for gestational age and FGR fetuses, showing an association between decreased ADC values in the frontal regions and adverse perinatal outcomes, while other brain regions were not significantly different[53]. However, the long-term implications of reduced ADC values in the fetal brain remains to be elucidated by long-term follow-up studies.

Lower ADC values in the central and peripheral placenta of FGR pregnancies have been reported [25], [54], [55], [56], [57]. This restricted diffusion might be a combination of acute and chronic hypoxic injuries as well as the prevalence of hematomas and infarctions. All these factors reduce blood supply and contribute to scarring and loss of cellular integrity of the placental tissue [54]. The central region of the placenta often shows higher perfusion and oxygenation due to the vascular architecture of the placenta, potentially making it less affected by hypoxic conditions [58]. *In vivo* assessment of placental health is not part of standard care when using ultrasound. Accurate monitoring and timing of delivery for early onset-FGR remains one of the biggest challenges; by adding this information provided with DWI we could potentially predict or optimize our timing of delivery based on placenta diffusion deterioration [59]. Additionally, T2* MRI holds promise for the future since it directly measures placenta oxygenation [58], [60].

In this prospective study a clear definition of early-onset brain-sparing FGR was implemented. Our data was compared to data from the YOUth cohort which included a significant number of women with uncomplicated pregnancies, addressing a critical limitation in literature where control groups often include participants undergoing fetal MRI for clinical reasons. By prospectively scanning participants in both the FGR and healthy control group using the same 3 T MRI system and scan protocol, we minimized bias.

A limitation is the small sample size for the FGR group, this might have led to a type I error[61]. A larger cohort would have allowed for subgroup analyses on severity of FGR, gender, ethnicity and maternal smoking. The significant differences in baseline characteristics are another limitation. Ethnicity is known to influence birthweight genetically [62]. Furthermore, the FGR group includes fewer female fetuses whereas sex-based differences in brain development have been established. Males generally have larger brains, yet our FGR group had fewer males compared to controls, potentially masking a larger difference. [63]. Future studies should aim to better match FGR and control groups or consider establishing collaborations or registries with different hospitals to address this limitation. Moreover, all analyses, including volumetric measurements and ROI placement for the ADC-values, should be conducted blinded to clinical condition to minimize bias, and ideally performed by multiple operators to allow assessment of interobserver reliability. Lastly, the interpretation of fetal brain differences measured with MRI in relation to the long-term neurodevelopmental outcome for the FGR awaits as follow-up is currently still ongoing. Those outcomes could aid in a better prediction for a personalized prognosis.

This study indicates that fetal MRI, using techniques like SVR fetal MRI segmentation and DWI, can detect altered brain development in FGR as early as 30 weeks of gestation. While the observed reductions in brain volumes and ADC values suggest potential chronic hypoxia, these findings should be approached with caution, particularly given the need to correct for the difference in GA between the FGR and control group. Furthermore, given that corticosteroids are routinely administered in early-onset FGR, our findings likely reflect brain development under typical clinical conditions. Nevertheless, we acknowledge that corticosteroid exposure may influence diffusion metrics and brain size through transient white matter changes, and this should be considered when interpreting the results [64], [65]. Further research is necessary to replicate our findings and to understand their implications fully. By employing such MRI measures, clinicians might be able to intervene earlier, potentially preventing more severe alterations in brain growth and volume. However, these possibilities remain speculative, requiring validation through larger studies that also consider potential confounders. This approach could eventually lead to more tailored treatments and improved outcomes in pregnancies affected by FGR.

## Author statement

LM segmented the FGR fetal scans. IvO segmented the YOUth fetal scans. RN assessed all MRI scans for structural anomalies. FL installed the fetal segmentation pipeline. LM analyzed all data and drafted the first version of the manuscript with the support of FT. All authors contributed to the interpretation of the results, critically revised the manuscript, and approved the final version. MB and MB supervised the study.

## CRediT authorship contribution statement

**Rutger A.J. Nievelstein:** Writing – review & editing, Methodology, Investigation. **Femke Lammertink:** Writing – review & editing, Software, Methodology. **Manon Benders:** Writing – review & editing, Supervision, Resources, Project administration, Data curation, Conceptualization. **Mireille Bekker:** Writing – review & editing, Supervision, Resources, Project administration. **Lotte Meijerink:** Writing – original draft, Project administration, Methodology, Investigation, Formal analysis, Data curation, Conceptualization. **Inge van Ooijen:** Writing – review & editing, Methodology, Investigation, Data curation, Conceptualization. **Fieke Terstappen:** Writing – review & editing, Supervision, Formal analysis, Data curation. **Thomas Alderliesten:** Writing – review & editing, Supervision, Conceptualization.

## Declaration of Competing Interest

The authors declare that they have no known competing financial interests or personal relationships that could have appeared to influence the work reported in this paper.

## Footnotes

Appendix A Supplementary data associated with this article can be found in the online version at doi:10.1016/j.eurox.2025.100417.
