# Supplementary material for: Fetal MRI study of brain differences in early-onset fetal growth restriction versus healthy controls at 30 weeks of gestation
Source: Eur J Obstet Gynecol Reprod Biol X. 2025 Jul 3;27:100417. doi: 10.1016/j.eurox.2025.100417 (PMC12275939; doi:10.1016/j.eurox.2025.100417)
Supplement: Supplementary file 1 — Supplementary material [file mmc1.docx]

**Table S1: Biometric and Doppler Data per Patient in the FGR cohort**

| Participant | GA at US | GA at MRI | Days of BS | Gender | HC (mm) | HC p | AC (mm) | AC p | FL (mm) | FL p | EFW (g) | EFW p | UA PI | UA PI p | UA EDF | MCA PI | MCA PI p | CPR | DV PI | Amniotic Fluid |
| --- | --- | --- | --- | --- | --- | --- | --- | --- | --- | --- | --- | --- | --- | --- | --- | --- | --- | --- | --- | --- |
| 1 | 31.71 | 31.43 | 6 | male | 261.3 | 0 | 201.7 | 0.00 | 46.2 | 0 | 823 | 0 | 1.36 | 97.3 | Positive | 0.92 | 0 | 0.68 | 0.46 | normal |
| 2 | 29.57 | 30.57 | 7 | male | 255.3 | 0.7 | 217.9 | 0.10 | 46.6 | 0 | 910 | 0.2 | 2.32 | 100 | Positive | 1.91 | 21.1 | 0.82 | 0.6 | normal |
| 3 | 28.71 | 29.14 | 11 | male | N/A | N/A | 205 | 0.00 | 44.6 | 0 | 768 | 0.1 | 1.1 | 60.5 | Positive | 1.46 | 1.2 | 1.33 | 1.01 | normal |
| 4 | 29.29 | 28.86 | 4 | male | 260.1 | 4.7 | 222 | 0.70 | 43.5 | 0 | 890 | 0.2 | 1.35 | 93.1 | Positive | 1.2 | 0.1 | 0.89 | 0.44 | normal |
| 5 | 31.14 | 31.14 | 7 | female | 264.8 | 0.3 | 238.7 | 0.80 | 55.8 | 9.1 | 1266 | 1.5 | 1.57 | 99.6 | Positive | 1.41 | 1.3 | 0.9 | 0.54 | normal |
| 6 | 29.86 | 29.57 | 1 | female | 255.3 | 0.3 | 236.8 | 4.50 | 52.8 | 5.6 | 1156 | 3.2 | 2.13 | 100 | Reversed | 1.43 | 1.1 | 0.67 | 1.02 | normal |
| 7 | 31.86 | 31.14 | 2 | female | 278.6 | 3.1 | 244.3 | 0.80 | 55.4 | 2.1 | 1344 | 1.2 | 2.22 | 100 | Reversed | 1.19 | 0.1 | 0.54 | 0.51 | normal |
| 8 | 31.14 | 31.86 | 5 | male | 286 | 29.7 | 241.1 | 1.30 | 54.5 | 2.9 | 1321 | 2.6 | 1.42 | 98.1 | Positive | 1.23 | 0.2 | 0.87 | 0.38 | normal |
| 9 | 28.57 | 29.43 | 6 | male | 251.2 | 2.4 | 220.6 | 2.00 | 45.3 | 0 | 893 | 0.9 | 1.66 | 99.4 | Positive | 1.2 | 0 | 0.72 | 0.62 | normal |
| 10 | 26.14 | 26.00 | 27 | male | 227.9 | 2.2 | 181.9 | 0.00 | 39.4 | 0 | 569 | 0.2 | 1.4 | 87.5 | Positive | 1.86 | 17.6 | 1.33 | 1.06 | normal |
| 11 | 31.57 | 31.57 | 7 | female | 285.5 | 17.4 | 232.8 | 0.10 | 56.8 | 10.8 | 1311 | 1.3 | 1.37 | 97.4 | Positive | 1.31 | 0.6 | 0.96 | 0.75 | normal |
| 12 | 27.57 | 27.57 | 2 | male | 232.7 | 0.1 | 197.3 | 0.10 | 40.6 | 0 | 660 | 0.1 | 1.43 | 93.4 | Positive | 1.06 | 0 | 0.74 | 0.47 | normal |
| 13 | 30.86 | 31.14 | 4 | male | 263.4 | 0.3 | 214.3 | 0.00 | 48.1 | 0 | 934 | 0 | 1.58 | 99.5 | Positive | 1.14 | 0 | 0.72 | 0.56 | normal |
| 14 | 27.57 | 28.00 | 3 | male | 253.1 | 25.9 | 207.7 | 0.90 | 46.2 | 0.7 | 839 | 2.3 | 2 | 99.99 | Absent | 1.62 | 4.1 | 0.81 | 0.69 | normal |
| 15 | 27.57 | 27.57 | 3 | male | 242.3 | 2.6 | 209 | 1.30 | 43.5 | 0 | 780 | 0.8 | 2.35 | 100 | Reversed | 1.9 | 18.6 | 0.81 | 1.1 | normal |
| 16 | 29.00 | 29.00 | 4 | male | 252.5 | 1.2 | 211.7 | 0.10 | 42.5 | 0 | 797 | 0.1 | 1.11 | 64.2 | Positive | 1.01 | 0 | 0.91 | 0.43 | normal |
| 17 | 31.86 | 32.00 | 12 | male | 273.1 | 0.7 | 227.6 | 0.00 | 50.7 | 0 | 1096 | 0.1 | 2.07 | 100 | Positive | 1.67 | 11.5 | 0.81 | 0.68 | normal |
| 18 | 31.14 | 31.43 | 11 | male | N/A | N/A | 237.6 | 0.60 | 51.4 | 0.1 | 1165 | 0.5 | 1.6 | 99.7 | Reversed | 1.1 | 0 | 0.69 | 0.81 | normal |
| 19 | 29.14 | 29.14 | 8 | male | 247 | 0.1 | 182 | 0.00 | 38.9 | 0 | 591 | 0 | 2.24 | 100 | Absent | 2.66 | 82 | 1.19 | 0.77 | normal |
| 20 | 27.57 | 27.71 | 1 | female | 247.2 | 8.9 | 212 | 1.17 | 49.1 | 13.2 | 903 | 5.7 | 1.56 | 97.6 | Positive | 1.59 | 3.3 | 1.02 | 0.61 | normal |
| 21 | 29.14 | 28.86 | 1 | female | 241.2 | 0 | 214.7 | 0.20 | 45.3 | 0.2 | 838 | 0.1 | 1.77 | 99.8 | Absent | 1.69 | 6.9 | 0.95 | 1.03 | normal |
| 22 | 31.57 | 31.86 | 7 | female | 254.3 | 0 | 221.3 | 0.00 | 53.2 | 0.3 | 1057 | 0.1 | 1.52 | 99.4 | Positive | 1.26 | 0.3 | 0.83 | 0.15 | oligohydramnion |
| 23 | 30.14 | 30.86 | 18 | male | 257.8 | 0.3 | 218.4 | 0.00 | 49 | 0 | 963 | 0.2 | 1.95 | 100 | Absent | 1.22 | 0.1 | 0.63 | 1.07 | oligohydramnion |
| 24 | 26.29 | 26.29 | 8 | female | 232.5 | 5.4 | 184.8 | 0.00 | 39.9 | 0 | 595 | 0.2 | 1.65 | 98.1 | Positive | 1.42 | 0.9 | 0.86 | 0.82 | normal |
| 25 | 31.43 | 31.71 | 2 | female | 277.5 | 4.8 | 238.2 | 0.40 | 54.4 | 1.6 | 1269 | 1 | 1.81 | 100 | Absent | 1.42 | 1.6 | 0.78 | 0.47 | normal |
| 26 | 30.86 | 31.43 | 4 | male | 286.4 | 40.3 | 235 | 0.60 | 53.9 | 2.7 | 1260 | 2.2 | 1.96 | 100 | Absent | 1.31 | 0.4 | 0.67 | 0.99 | normal |
| **Mean** | **29.66** | **29.82** | **6.58** |  | **257.79** | **6.31** | **217.48** | **0.60** | **47.98** | **1.90** | **961.46** | **0.95** | **1.71** | **95.56** |  | **1.43** | **6.65** | **0.85** | **0.69** |  |

AC = abdominal circumference, BS = brain-sparing, CPR = cerebroplacental ratio, DV = ductus venosus, EDF = end diastolic flow, EFW = estimated fetal weight, FGR = fetal growth restriction, GA = gestational age, HC = head circumference, MCA = middle cerebral artery, MRI = magnetic resonance imaging, N/A = not available, measurement failed, p = percentile, PI = pulstatility index, UA = umbilical artery, US = ultrasound

**Table S2: Absolute regional brain volumes (ml)**

| **Characteristics** | **FGR (N= 22)** | **Control (N= 59)** | **p-value** |
| --- | --- | --- | --- |
| eCSF total | 49.84 ± 12.50 | 85.16 ± 15.32 | <0.001 |
| Cortex left | 19.44 ± 1.80 | 22.60 ± 2.81 | <0.001 |
| Cortex right | 19.52 ± 1.49 | 22.43 ± 2.73 | <0.001 |
| Cortex total | 38.96 ± 3.19 | 45.02 ± 5.49 | <0.001 |
| WM left | 44.88 (4.81) | 49.60 (8.20) | <0.001 |
| WM right | 44.66 ± 3.66 | 50.97 ± 5.48 | <0.001 |
| WM total | 88.84 (8.49) | 99.46 (17.52) | <0.001 |
| Lateral ventricle left | 1.84 (1.45) | 2.53 (1.30) | 0.091 |
| Lateral ventricle right | 1.99 (1.17) | 2.30 (0.73) | 0.464 |
| Brainstem | 3.50 ± 0.27 | 3.95 ± 0.34 | <0.001 |
| Cerebellum left | 2.79 ± 0.29 | 3.34 ± 0.48 | <0.001 |
| Cerebellum right | 2.79 ± 0.33 | 3.41 ± 0.41 | <0.001 |
| Vermis | 0.91 ± 0.12 | 1.21 ± 0.14 | <0.001 |
| Cerebellum total | 6.49 ± 0.72 | 7.97 ± 0.98 | <0.001 |
| Lentiform left | 2.21 ± 0.23 | 2.52 ± 0.26 | <0.001 |
| Lentiform right | 2.17 ± 0.22 | 2.50 ± 0.26 | <0.001 |
| Thalamus left | 1.66 ± 0.14 | 1.80 ± 0.19 | 0.003 |
| Thalamus right | 1.61 (0.24) | 1.84 (0.23) | <0.001 |
| DGM | 7.65 ± 0.76 | 8.66 ± 0.82 | <0.001 |
| Third ventricle | 0.16 ± 0.04 | 0.19 ± 0.04 | <0.001 |
| Fourth ventricle | 0.10 (0.03) | 0.09 (0.02) | 0.779 |
| **TBV** | **144.24 ± 11.54** | **166.87 ± 17.51** | **<0.001** |

FGR = fetal growth restriction, eCSF = extracerebral spinal fluid, DGM = deep gray matter (sum of left and right lentiform nucleus and thalamic volume), TBV = total brain volume

**Table S3: – Post Hoc Power Analysis Of Observed Findings**

| **Variable** | **Test used** | **Effect size (g or δ) and 95% CI** | **Effect size intepretation** |
| --- | --- | --- | --- |
| Brain volumes | | | |
| Cortex | Hedges’ g | -1.22, 95% CI [-1.68; -0.80] | Large |
| White Matter | Cliff’s Delta | -0.66, 95% CI [-0.82; -0.46] | Large |
| Brainstem | Hedges’ g | -1.44, 95% CI [-2.07;-0.91] | Large |
| Cerebellum | Hedges’ g | -1.63, 95% CI [-2.24; -1.13] | Large |
| Deep Gray Matter | Hedges’ g | -1.27, 95% CI [-1.84;-0.78] | Large |
| Total Brain Volume | Hedges’ g | -1.41, 95% CI [-1.84; -1.03] | Large |
| Cortex/ TBV | Cliff’s Delta | -0.05, 95% CI [-0.33;-0.25] | Negligible |
| White Matter/ TBV | Cliff’s Delta | 0.13, 95% CI [-0.17;0.42] | Negligible |
| Brainstem/ TBV | Cliff’s Delta | 0.13, 95% CI [-0.14; 0.40] | Negligible |
| Cerebellum/ TBV | Cliff’s Delta | -0.40, 95% CI [-0.62;0.16] | Medium |
| Deep Gray Matter/ TBV | Cliff’s Delta | 0.20, 95% CI [-0.11;0.49] | Small |
| ADC-values | | | |
| FWM | Hedges’ g | -0.71, 95% CI [-1.40; -0.10] | Medium |
| OWM | Hedges’ g | -1.00, 95% CI [-1.60; 0.48] | Large |
| CSO | Hedges’ g | 0.40, 95% CI [-1.09; 0.89] | Small |
| Thalami | Cliff’s Delta | -0.50, 95% CI [-0.80; 0.17] | Large |
| CBH | Cliff’s Delta | 0.14, 95% CI [-0.20; 0.48] | Negligible |
| Pons | Cliff’s Delta | -0.43, 95% CI [-0.71;0.14] | Medium |
| Central placenta | Cliff’s Delta | -0.87, 95% CI [-1.00; -0.65] | Large |
| Peripheral placenta | Cliff’s Delta | -0.87, 95% CI [-0.99;-0.69] | Large |

ADC = apparent diffusion coefficients, CBH = cerebellar hemispheres, CI = confidence interval, CSO = centrum semi ovale, FWM = frontal whit matter, OWM = occipital white matter, TBV = total brain volume
